# YOLO-BFRV: An Efficient Model for Detecting Printed Circuit Board Defects

**DOI:** 10.3390/s24186055

**Published:** 2024-09-19

**Authors:** Jiaxin Liu, Bingyu Kang, Chao Liu, Xunhui Peng, Yan Bai

**Affiliations:** College of Computer and Control Engineering, Northeast Forestry University, Harbin 150040, China; 13194202459@163.com (J.L.); 18072424470@163.com (B.K.); 18304624155@163.com (C.L.); 13979472525@163.com (X.P.)

**Keywords:** PCB defect detection, YOLOv8, BIFPN, FasterNet, RepHead, loss function

## Abstract

The small area of a printed circuit board (PCB) results in densely distributed defects, leading to a lower detection accuracy, which subsequently impacts the safety and stability of the circuit board. This paper proposes a new YOLO-BFRV network model based on the improved YOLOv8 framework to identify PCB defects more efficiently and accurately. First, a bidirectional feature pyramid network (BIFPN) is introduced to expand the receptive field of each feature level and enrich the semantic information to improve the feature extraction capability. Second, the YOLOv8 backbone network is refined into a lightweight FasterNet network, reducing the computational load while improving the detection accuracy of minor defects. Subsequently, the high-speed re-parameterized detection head (RepHead) reduces inference complexity and boosts the detection speed without compromising accuracy. Finally, the VarifocalLoss is employed to enhance the detection accuracy for densely distributed PCB defects. The experimental results demonstrate that the improved model increases the mAP by 4.12% compared to the benchmark YOLOv8s model, boosts the detection speed by 45.89%, and reduces the GFLOPs by 82.53%, further confirming the superiority of the algorithm presented in this paper.

## 1. Introduction

Printed circuit boards (PCBs) are extensively used in the automotive and communications sectors as well as numerous other critical modern industrial sectors. During the PCB production process, which involves pressing, drilling, copper deposition, dry film application, and other processes [1], defects may be introduced at each stage. Consequently, PCB defect detection technology has become a critical quality control technology in the electronics manufacturing industry. As the world progresses towards digitization, PCB design and production increasingly focus on miniaturization, high precision, high density, and high reliability. Traditional methods, such as manual inspection [2], electrical detection [3], and automatic optical inspection [4], are becoming insufficient to meet the current requirements of PCB manufacturing. Image processing techniques, including threshold segmentation [5], edge detection [6] and region growth [7] methods, are constrained by challenges such as feature selection, lighting conditions, and noise interference. These methods often necessitate manual rules and feature definitions, leading to algorithms with relatively low robustness and generalization capabilities. This undoubtedly presents a formidable challenge to deep learning-based PCB defect detection.

The intricate manufacturing process of PCBs frequently generates various types of defects, including rat bites, spurs, and open circuits. Even minor imperfections can significantly impact PCB performance, posing substantial challenges for models to identify multiple defects with efficiency and accuracy simultaneously. In this regard, Xia et al. [8] proposed the SSIM-Net network, which employs a lightweight backbone network, MobileNet-V3, to classify defective regions, dramatically increasing the speed of PCB defect detection without losing accuracy. Liu and Wen [9] proposed a PCB defect detection model (MobileNet-YOLO-Fast) with a small model size and good real-time performance. Owing to the varying manufacturing processes, PCB surface defects typically measure less than 4500 pixels, with some, such as spurs, being smaller than 300 pixels. Consequently, detecting these small-scale defects swiftly and accurately becomes exceedingly challenging. Ding et al. [10] proposed TDD-Net, which uses the k-means++ clustering method to obtain more accurate anchor frames and efficiently detect minor target defects. In 2023, Lim et al. [11] addressed the difficulty of inferring small or changing defects on PCBs in real time by enhancing tiny defect detection through contextual information inclusion. Achieving high accuracy in defect detection models necessitates a substantial amount of data. However, when the available PCB product image data are insufficient, the model struggles to learn and distinguish the features of various defects effectively. Hua [12] designed a recursive residual network to aggregate the features extracted by this model to obtain the indicators reflecting the target features to improve detection accuracy. Park et al. [13] proposed the MarsNet detection network, which solves the problem of different scales of actual defect data and training data by improving the expanded residual network.

By addressing the aforementioned challenges while integrating the inherent physical characteristics of PCB defects, this paper proposes a PCB defect detection method that improves the YOLOv8 model. The main contributions of this study are summarized as follows:

(1) The introduction of a BIFPN in the Neck addresses the problems of PANet in the original model regarding the fusion of diverse layer features. This enhancement significantly improves the model’s capacity to capture small target feature information, thereby enabling more efficient extraction of various defect features.
(2) The FasterNet lightweight network is integrated into the Backbone, effectively utilizing the channel information across various PCB defect types. This integration preserves the diversity of defect features and enhances the computational efficiency all while improving detection accuracy.
(3) The detection head has been reconstructed, incorporating a re-parameterized design that reduces the number of parameters while enhancing the detection capabilities for small target defects.
(4) To address the challenge of detecting PCB defects in dense distributions, this paper employs VarifocalLoss as the loss function. This approach mitigates the difficulties associated with defect detection in such contexts and significantly enhances the model’s detection accuracy.

The remainder of this paper is organized as follows: Section 2 outlines the creation of the dataset and the improved model with four modules. Subsequently, Section 3 presents a thorough analysis of the experimental results. Finally, Section 4 offers a summary of the contributions and the limitations of this study.

## 2. Materials and Methods

### 2.1. Dataset

#### 2.1.1. Dataset Collection

PCB defect detection technology is mainly used for its image processing abilities to identify defects, and the efficacy of a detection model is largely contingent upon the quality of the dataset used. At present, various standards exist for PCB defects. The IPC standard, established by the U.S. Electronics Industry Connection Association, has emerged as a widely recognized benchmark within the printed circuit board industry. IPC standards [14], known for their adaptability and versatility, are extensively employed in the electronics sector to foster standardization and safety in electronic assembly while also offering critical technical guidance. This study utilizes the HRIPCB [15] dataset, publicly released by Peking University, which adheres to the IPC standard. The HRIPCB dataset comprises 1386 images of PCB defects, categorized into six types: missing_hole, mouse_bites, open circuits, short, spur, and spurious_copper.

#### 2.1.2. Dataset Augmentation

**Figure 1:**
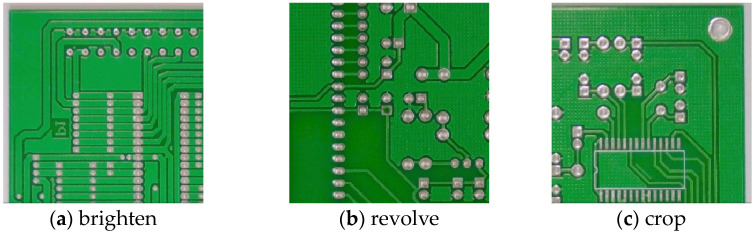
Results of augmentation. (**a**) Image after brightening; (**b**) image after revolving; (**c**) image after cropping.

Given the small sample size of this dataset, overfitting is a potential issue during model training. To address this issue, the dataset is augmented through highlighting, rotating, and cropping techniques, as illustrated in Figure 1. These data enhancement strategies aim to improve the model’s performance. Following data enhancement, the initial dataset, comprising 1386 images, is increased to 10,668 images. And the total number of different types of defects in the expanded dataset reaches 19,854.

### 2.2. Methods

#### 2.2.1. Bidirectional Feature Pyramid Network (BIFPN)

**Figure 2:**
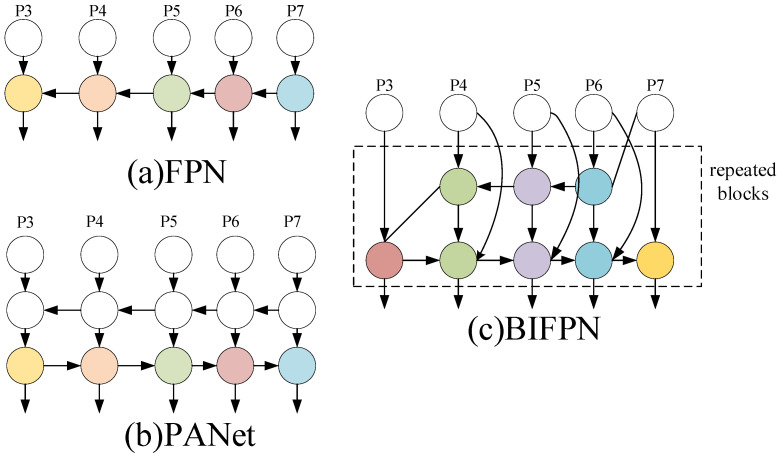
Structure of FPN and BIFPN.

The desire for precise and rapid defect identification in PCB detection scenarios necessitates more advanced feature fusion mechanisms. YOLOv8 still adopts the idea of the path aggregation network (PANet [16]) structure within the Neck, as illustrated in Figure 2b. In contrast to the unidirectional, top-down feature pyramid network (FPN) [17] structure shown in Figure 2a, PANet is a bidirectional pathway network. It incorporates a bottom-up path in addition to the FPN, resulting in more efficient feature propagation. However, PANet networks demand greater computational resources, leading to relatively slower processing speeds and suboptimal performance for real-time target detection tasks. Furthermore, PANet utilizes a bottom-up path aggregation network to improve information flow. This approach may degrade detection accuracy if the low-level feature information is insufficient or partially lost [18].

Based on previous research on feature fusion, Mingxing Tan et al. from Google’s BrainTeam proposed a novel architecture, EfficientDet, which newly proposes an efficient weighted bidirectional feature pyramid network (BiFPN) [19] in the Neck. The BIFPN introduces learnable weights to assess the significance of various input features and performs multi-scale feature fusion efficiently. This is achieved through the following strategies (the structure of the BiFPN is depicted in Figure 2c): Firstly, the bidirectional network is simplified by eliminating nodes with input edges that do not contribute to feature fusion, thereby enhancing the feature network’s fusion process. Secondly, an additional edge is introduced between the input and output nodes to address scenarios where both are located in the same layer. Finally, an adaptive weighting mechanism is employed, enabling the model to learn the contribution of each input feature based on the learned weights, thereby achieving more accurate PCB defect detection results.

#### 2.2.2. FasterNet

**Figure 3:**
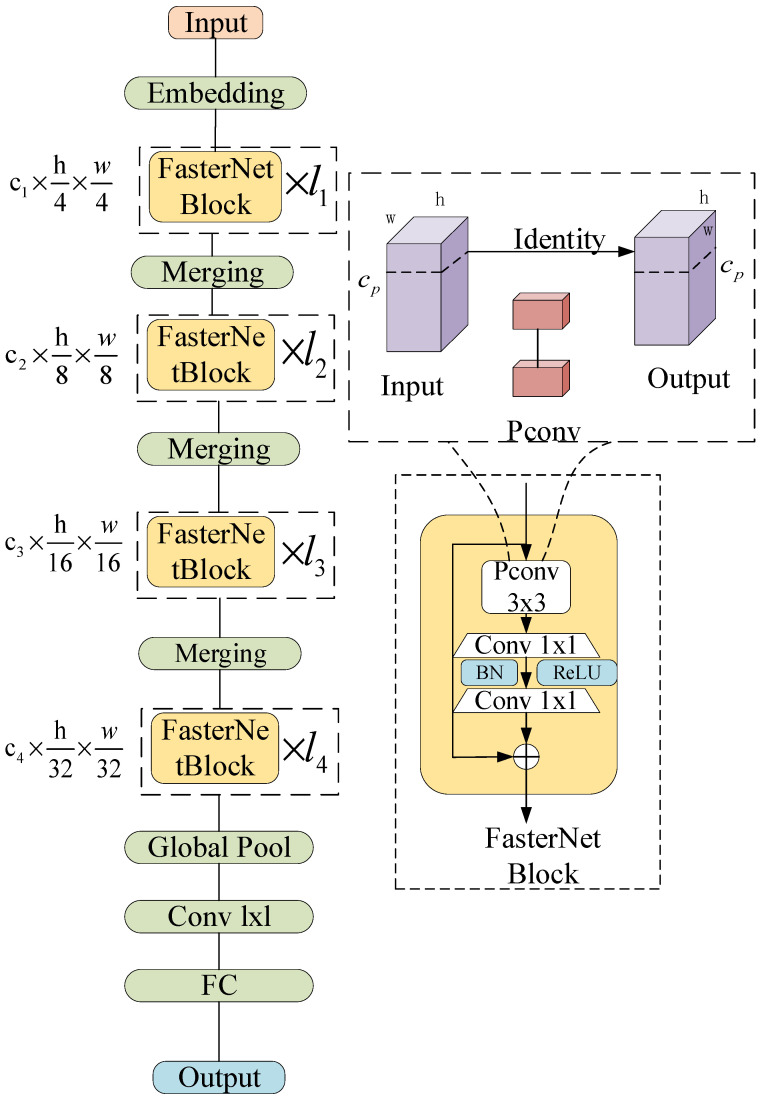
FasterNet structure.

To fulfill the stringent demands of PCB defect detection, particularly in terms of accuracy and real-time performance, this study substitutes the CSPDarkNet [20] feature extraction network in YOLOv8 with FasterNet, significantly reducing the model’s computational complexity while enhancing the detection speed. FasterNet, proposed by Chen et al. [21] in 2023, is a lightweight network designed with the core principle of enhancing feature representation capabilities while preserving a high speed and lightweight characteristics. The structure of FasterNet is illustrated in Figure 3. The primary architecture of FasterNet comprises four stages, each containing a FasterNet Block, which includes a PConv layer followed by two 1 × 1 convolutional layers, with an embedding or merging layer being positioned before each convolutional layer. Compared with the backbone feature extraction network of YOLOv8, FasterNet uses a new convolution operator, PConv (partial convolution), as well as PWConv (point-by-point convolution) as the principal operator. This results in enhanced detection accuracy and speed, alongside reductions in memory access and computational redundancy. The FasterNet Block serves as the fundamental building module of this network. It features an inverted residual structure, where the middle PWConv expands channels and leverages shortcut connections to reutilize input features. Subsequently, batch normalization (BN) and the activation layer (ReLU) are integrated between the two PWConvs to preserve feature diversity and minimize latency. Finally, a global average pooling layer, followed by a 1 × 1 convolutional layer and a fully connected layer, is employed for feature transformation and classification. Partial convolution selectively performs convolution operations on a subset of input channels, leaving the remaining channels untouched. The floating-point calculation for the PConv component is presented in Equation (1):

(1) $$\mathrm{FLOPs}=h\times w\times k^{2}\times {c_{p}}^{2}$$

where h is the height of the channel, w is the width of the channel, k is the filter, and $c_{p}$ is a continuous network channel.

Compared to the backbone feature extraction network of YOLOv8, the lightweight FasterNet, designed with PConv and PWConv architectures, enhances the detection accuracy and speed while simultaneously minimizing memory access and computational redundancy, thereby enabling rapid and efficient PCB defect detection.

#### 2.2.3. RepHead Based on Structural Re-Parameterization Construction

**Figure 4:**
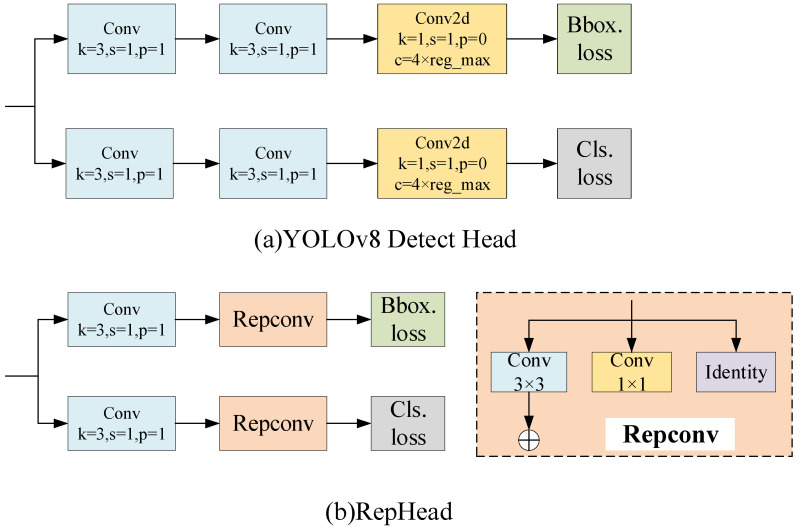
Structure of YOLOv8 detect head and RepHead.

When detecting small defects on PCB surfaces, the performance of the detection head is pivotal to the accuracy and efficiency of the model. An effective detection head can extract target information from input features more accurately, leading to more accurate predictions. To this end, we introduce a re-parameterization method. RepVGG [22] converts trained multi-branch models into single-branch inference models by utilizing re-parameterization techniques. Building upon this concept, we design a re-parameterization structure named RepConv and propose an efficient detection head termed RepHead [23]. A comparison between its structure and the YOLOv8 detector head is presented in Figure 4. The original model’s 3 × 3 convolution and 1 × 1 convolution are amalgamated into a new module, RepConv, through re-parameterization. During the training phase, RepConv employs a multi-branch structure, which facilitates the network’s learning and enhances its expressive capacity. This innovative method addresses the issues of feature sparsity and information loss due to the small sizes of defects on the PCB surface, thereby enabling the model to identify these defects more effectively. It reduces the model’s parameter count while enhancing the detection accuracy and significantly improving the detection speed.

#### 2.2.4. Varifocal Loss

Since PCB defects predominantly consist of small targets of varying sizes, YOLOv8 uses the Binary CrossEntropYLoss (BCELoss) [24] as the classification loss function to mitigate the impact of class imbalance during the training process, which is defined by Equation (2):

(2) $$BCEL(p,y)=\left\{\begin{array}{l} -\log(p)\;\;\;if\;y=1\\ -\log(1-p)\;\;\;\mathrm{otherwise} \end{array}\right.$$

where p is the predicted label and y is the true label.

Given that the dense detector must analyze every position within an image, positive samples (detection targets) constitute only a minute fraction of the total, with the majority being negative samples (background). The overwhelming number of negative samples dominates the gradient contribution process, thereby affecting the model’s capacity for accurate predictions. Focal Loss [25] addresses the imbalance in the handling of positive and negative samples by introducing a weighting factor α and a modulation factor, enabling the model to focus more on detecting densely distributed and difficult-to-detect samples during training, as defined by Equation (3):

(3) $$FL(p,y)=\left\{\begin{array}{l} -\alpha {\left(1-p\right)}^{y}\;\log(p)\;\;\;\mathrm{if}\;y=1\\ -(1-\alpha )p^{\gamma }\;\log(1-p)\;\;\;\mathrm{otherwise} \end{array}\right.$$

However, the above factors primarily reduce the loss of easily classifiable samples without fully leveraging the gradient contributions from both positive and negative samples. Given that the current model relies on both target classification information and location to achieve optimal results, we employ Varifocal Loss [26], which not only dynamically adjusts the weights of negative samples to balance their overwhelming impact on predictions but also preserves the extraction of positive samples. This enhances the model’s learning capacity and achieves superior PCB defect detection performance. Varifocal Loss is defined through Equation (4):

(4) $$VFL(p,y)=\left\{\begin{array}{l} -q(q\log(p)+(1-q)\log(1-p)\;\;q>0\\ -\alpha p^{\gamma }\;\log(1-p)\;\;\;q=0 \end{array}\right.$$

where p is the perceptual classification score (IACS) [27], q is the object score, γ is the modulation factor, and α is the weighting factor.

#### 2.2.5. YOLO-BRFV Model

**Figure 5:**
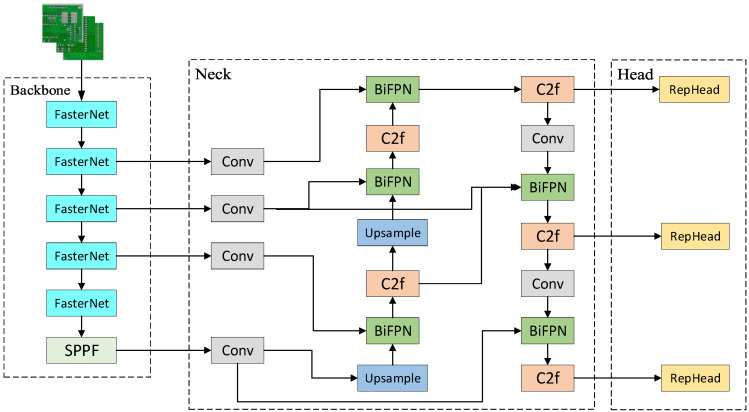
YOLO-BFRV network model structure.

Based on the analyses in Section 2.2.1, Section 2.2.2, Section 2.2.3 and Section 2.2.4, we present the improved model of this study, with its structure illustrated in Figure 5. First, the BIFPN is incorporated into the Neck layer, replacing the original PANet, which enhances the algorithm’s target detection and classification performance. Second, the lightweight FasterNet is integrated into the Backbone, replacing the CSPDarkNet in the original model, thereby reducing computational delay and improving efficiency while enhancing detection accuracy. Additionally, this study reconstructs the re-parameterized detection head, replacing the original Head, thereby enhancing the model’s ability to capture small target details. Finally, Varifocal Loss is employed to replace the original loss function, significantly enhancing the detection accuracy for densely distributed defects.

### 2.3. Experimental Parameters and Environment

The experiment utilizes the Pytorch 1.3.0 framework and CUDA version 11.3 for training, with YOLOv8 serving as the base model, as shown in Table 1.

The configuration of hyperparameters significantly impacts the network’s prediction outcomes. Hyperparameters are values predefined before neural network training and are not derived from the sample data, meaning they are not data driven. This study evaluates the impact of hyperparameters and related strategies on model performance, including the batch size, initial learning rate, learning strategy, input image resolution, and optimizer choice.

For the batch size, we compared values of 8, 32, and 64 on the test set, ultimately determining that a batch size of 8 yielded the best model performance.

The learning rate greatly influences model convergence and performance, and its configuration is closely tied to the stability of the model during training. After a comparison, we set the initial learning rate to 0.01.

A higher input image resolution generally results in better model performance; however, it also increases the training iteration time. Considering this trade-off, the chosen input image resolution for this study is 640 × 640.

With a learning rate of 0.01, a batch size of 8, and an input image resolution of 640 × 640, a comparison between the SGD optimizer and Adam (Adaptive Moment Estimation) [28] optimizer revealed that the model failed to converge using SGD. Therefore, this study adopts the Adam optimizer.

During the training process, a training cycle (epoch) represents one complete pass of the dataset through the neural network. Typically, a total of 200 training cycles is used for small- to medium-sized datasets, yielding optimal results in most cases.

### 2.4. Model Evaluation Metrics

To comprehensively verify the effectiveness of the PCB defect detection model, the mean Average Precision (mAP) is commonly used as the evaluation metric. The mAP is calculated using precision (P) and recall (R), as shown in Equations (5) and (6).

(5) $$P=\frac{TP}{TP+FP}$$

(6) $$R=\frac{TP}{TP+FN}$$

In multi-class target detection, the average precision (AP) for each target class is typically calculated separately and then averaged to obtain the model’s mAP, as illustrated in Equations (7) and (8). The mAP value ranges from 0 to 1, with values closer to 1 indicating better model performance and higher detection accuracy. The detection speed of a model is typically measured in frames per second (FPSs), indicating the number of frames detected per second, as shown in Equation (9).

(7) $$AP=\int_{0}^{1}P\left(R\right)dR$$

(8) $$mAP=\frac{\sum_{i=1}^{k}AP_{i}}{k}$$

(9) $$FPS=\frac{Framenum}{ElapsedTime}$$

## 3. Results

### 3.1. Comparison of Different Benchmark Models

#### 3.1.1. Experimental Results of HIRIPCB

Current target detection algorithms are primarily categorized into one-stage and two-stage algorithms. Two-stage target detection algorithms, like R-CNN [28], SPN-Net [29], Fast R-CNN [30] and FasterR-CNN [31], rely on candidate regions, while one-stage target detection algorithms, such as YOLO [32] and SSD [33], are based on regression. Two-stage target detection algorithms require a series of candidate regions to be generated. Although they offer high accuracy, their detection speed is slow, making real-time detection tasks challenging. In contrast, one-stage algorithms bypass the candidate region generation step, resulting in faster detection speeds but poorer performance in detecting small targets. Given that PCB defect detection must satisfy both accuracy and real-time requirements, this study enhances the detection accuracy and speed by refining the YOLOv8 model. While maintaining the same training platform, environment, and dataset, we compare the experimental results of FasterR-CNN from the two-stage algorithm and YOLOv5, YOLOv7 [34], and YOLOv8s from the one-stage algorithm YOLO series. Additionally, we added the RT-DETR [35] algorithm for comparison, which is more effective in target detection. The experimental results are presented in Table 2. 

Compared to Faster-RCNN, the improved model in this study increases precision by 10.9 percentage points, recall by 5.9 percentage points, and mAP by 16.4% and reduces the number of parameters by 72%. Additionally, the model’s mAP improves by 2.9% compared to the RT-DETR algorithm while significantly reducing the number of parameters. Regarding the YOLO series algorithms, the improved model in this study also achieves strong results compared to YOLOv5, YOLOv7, and YOLOv8s, with the mAP values increasing by 6.3%, 3.9%, and 4.1%, respectively. The experimental results indicate that current models for PCB defect detection possess limitations, often resulting in misdetections and omissions. In this study, the improved YOLOv8 model introduces the BIFPN to strengthen feature extraction capabilities and employs Varifocal Loss to further enhance small target detection. In terms of parameter count, the model in this study reduces parameters by 78% compared to the benchmark model due to the use of PConv in FasterNet and RepConv in RepHead. Overall, the improved YOLOv8 model proposed in this study demonstrates robust comprehensive performance, enhancing the defect detection accuracy while maintaining an efficient detection speed, thereby providing a solid foundation for PCB defect detection in industrial applications.

**Figure 6:**
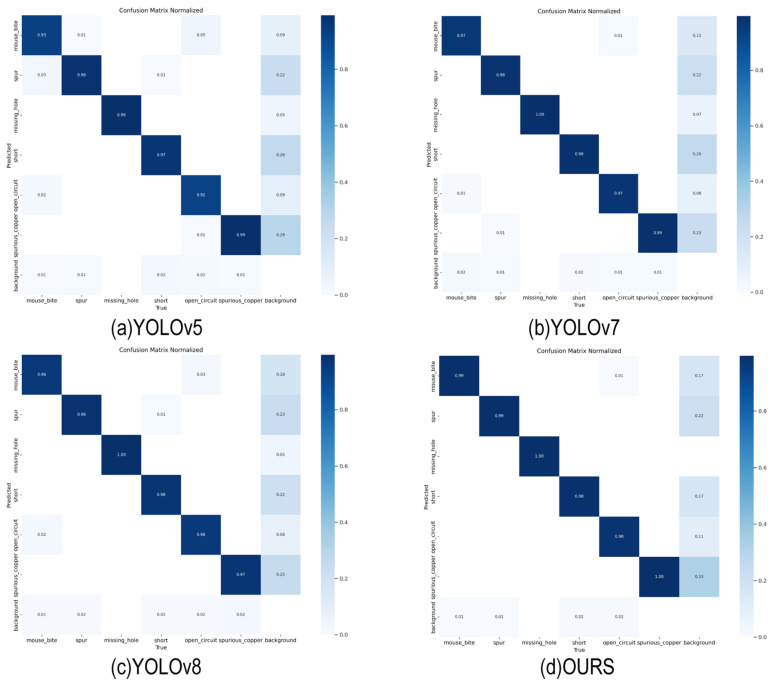
Confusion matrices for (**a**) YOLOv5; (**b**) YOLOv7; (**c**) YOLOv8; and (**d**) OURS.

Figure 6 compares the confusion matrices of the YOLO series algorithms. The diagonal elements represent the number of samples correctly predicted by the model, while the off-diagonal elements represent the number of incorrectly detected or missed samples. As observed in the figure, missing_hole and spurious_copper exhibit relatively high detection rates compared to the other categories, whereas short and spur exhibit higher rates of missed detections, demonstrating less effectiveness in comparison.

**Figure 7:**
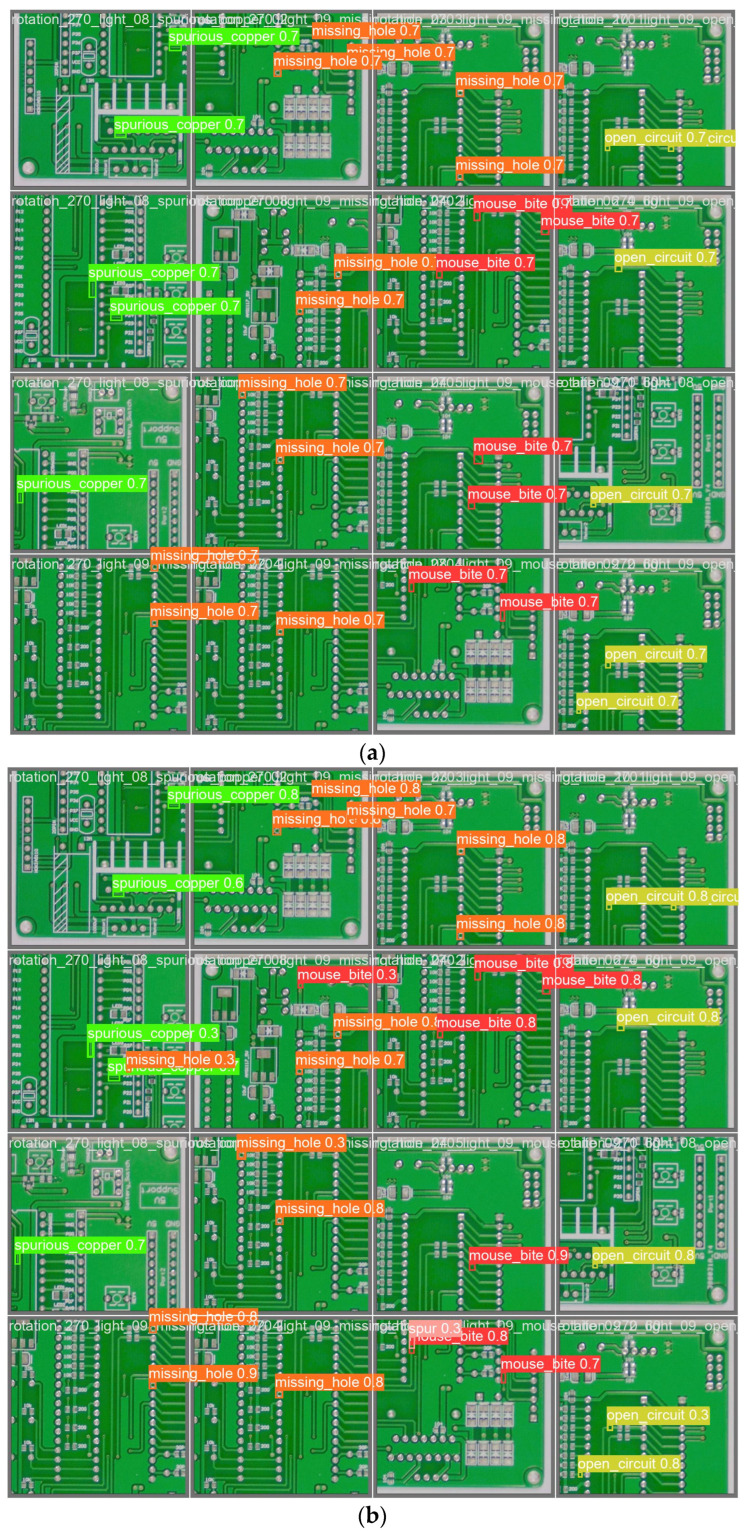

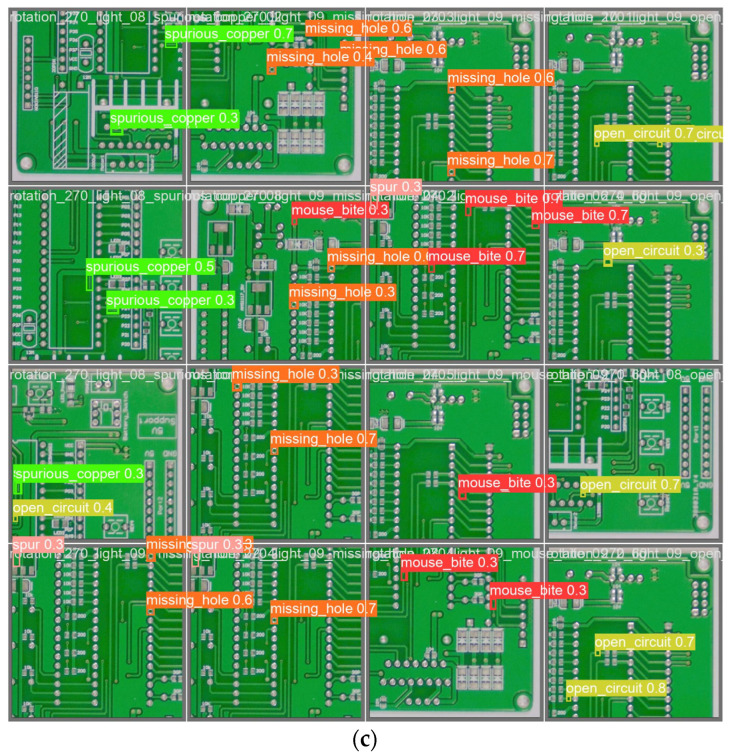
The detection results of the different models. (**a**) The results of the YOLOv8s model; (**b**) the results of the RT-DETR model; and (**c**) the results of the YOLO-BFRV model.

Figure 7 provides a detailed comparison of the detection outcomes between the proposed model and various algorithms discussed in this study. It is apparent that the benchmark model, YOLOv8s, faces considerable difficulties with false positives and missed detections. Specifically, the two defects—mouse bites and circuit openings—share similar visual characteristics, which heightens the risk of erroneous detections. This study addresses this issue by employing the BIFPN, which substantially improves the detection accuracy for visually similar targets. Moreover, the features of spur defects are particularly difficult to distinguish from the background. To address this challenge, Varifocal Loss is introduced, which adaptively adjusts the Focal Loss according to the complexity of the detection task. The model proposed in this study demonstrates superior effectiveness in detecting minute defects and exhibits an enhanced detection speed in comparison to the recently introduced target detection algorithm, RT-DETR.

#### 3.1.2. Experimental Results of DeepPCB

**Figure 8:**
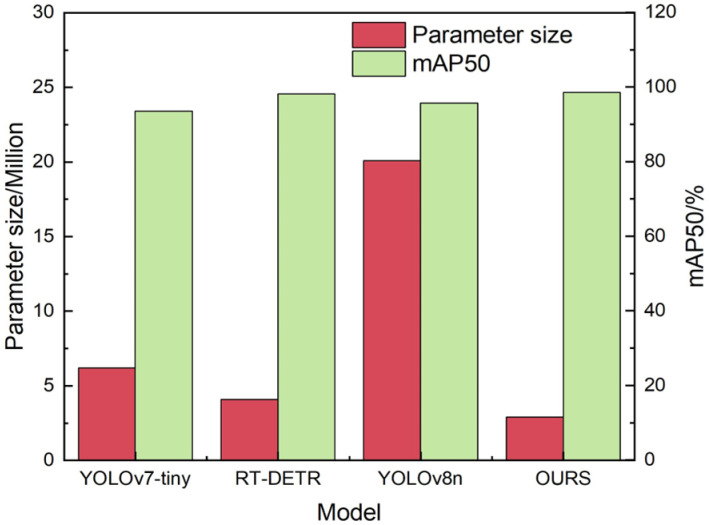
Experimental results of different models.

Additionally, relying on a single dataset is insufficient to assess the model’s generalization capability. Therefore, we extended our evaluation by incorporating another publicly available black-and-white image dataset, DeepPCB [36]. The images were captured using linear scanning Charge-Coupled Devices (CCDs), resulting in a dataset comprising 1500 images, each with a resolution of 640 × 640 pixels. The defect types in this dataset are consistent with those in the HRIPCB dataset, encompassing six distinct defect categories. Experiments were conducted utilizing the YOLOv8n, YOLOv7-tiny, RT-DETR, and YOLO-BFRV algorithms, with the corresponding results presented in Figure 8.

**Figure 9:**
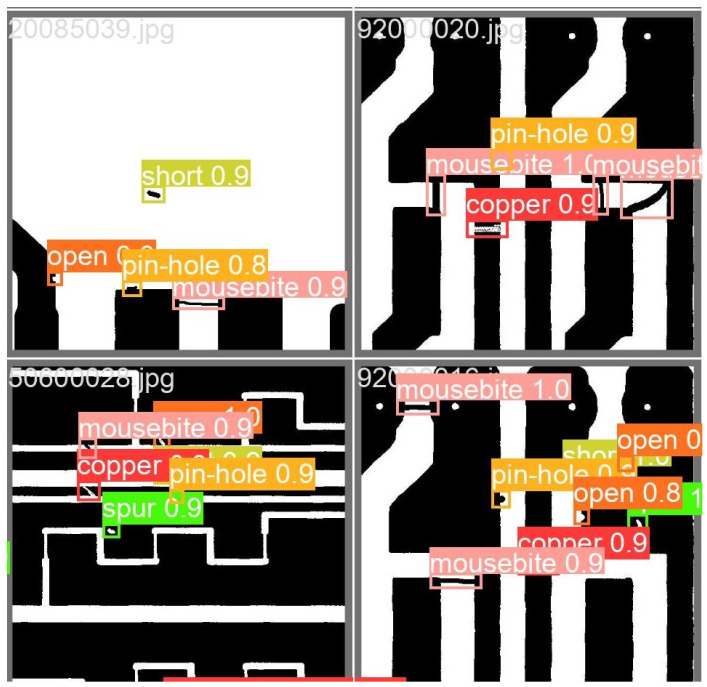
YOLO-BFRV defect detection results.

As shown in Figure 8, the YOLO-BFRV model achieves a mean Average Precision (mAP) of 98.6% on the DeepPCB dataset, representing improvements of 5.4%, 0.4%, and 3% over the YOLOv7-tiny, RT-DETR, and YOLOv8n models, respectively. The model comprises 2.9 million parameters, which is significantly smaller than other models, reflecting a 29% reduction compared to the baseline model. These results demonstrate that the YOLO-BFRV algorithm is not only effective with the HIRIPCB dataset, showcasing superior detection capabilities, but also performs robustly with the DeepPCB dataset, further validating the efficacy of the proposed algorithm. Selected test results from the improved model are presented in Figure 9.

### 3.2. Results of Ablation Experiments

To comprehensively evaluate the proposed model’s validity and feasibility, the performance of each component was rigorously assessed through ablation experiments. Given that the proposed model in this study is based on YOLOv8s, the YOLOv8s model was utilized as the benchmark for conducting the ablation experiments. The experiments utilized precision (P), recall (R), mean Average Precision (mAP), frames per second (FPS), and the total number of model parameters (GFLOPS) as evaluation metrics. The corresponding experimental results are presented in Table 3.

The following conclusions were derived from the ablation experiments:

(1) The benchmark model exhibits the lowest detection accuracy due to the presence of spurs, spurious_copper, and other small target defects within the dataset, which are challenging to identify, leading to a lower overall detection accuracy.
(2) Model C achieves a significant boost in detection speed after upgrading the backbone to FasterNet. However, the computational load is slightly increased due to the inclusion of PConv in its architecture. Models B, D, and E show varying degrees of improvement in detection accuracy and speed after the integration of sub-modules.
(3) Models F, G, and H, which incorporate BIFPN into the original architecture, demonstrate higher accuracy and a slight increase in detection speed compared to the traditional PANet structure in the original Neck component.
(4) After integrating all of the improved modules, Model I shows enhanced feature extraction and fusion capabilities by effectively suppressing irrelevant information. Consequently, the overall performance of the model surpasses that of the baseline, particularly in detecting smaller targets.

### 3.3. Loss Function Comparison Experiments

**Figure 10:**
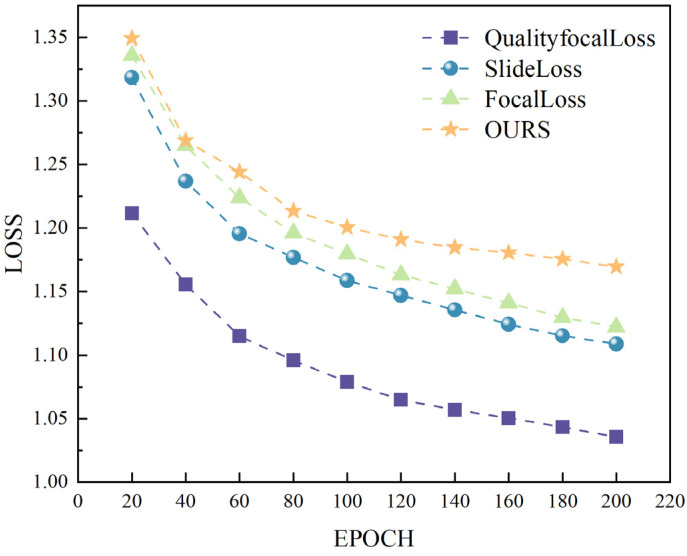
Experimental results of loss function comparison.

Varifocal Loss adjusts the contribution of negative samples to the loss without diminishing the loss of positive samples during training, thereby enhancing the generalizability and stability of the PCB defect detection model, which, in turn, improves the training accuracy. Focal Loss is particularly suitable for tasks with category imbalance, especially in scenarios with significant disparities between object and background ratios in target detection. It primarily addresses the imbalance between positive and negative samples by adjusting their weights as well as the weights of hard-to-classify samples. SlideLoss [37] employs a sliding window-based strategy that focuses on difficult defect samples, particularly those located at boundaries during training. This approach is mainly suitable for tasks requiring fine-grained, pixel-level classification, such as semantic segmentation, where it excels in boundary processing. Quality Focal Loss [38] enhances Focal Loss by integrating classification scores with the IoU of detection frames and transforming the target labels into continuous values. It adjusts the classification weights of both difficult and easy samples, making it particularly effective for improving the prediction quality. To further validate the superiority of Varifocal Loss, comparative experiments were conducted using Slide Loss, Focal Loss, and Quality Focal Loss. The experimental results, presented in Table 4, were obtained using mAP50%, Precision (P), and Recall (R) as performance metrics, with their comparisons being illustrated in Figure 10. The results demonstrate that Varifocal Loss, as utilized in this study, achieves the highest accuracy.

Based on the comparative experiments of the loss functions, the following conclusions can be drawn:

(1) As illustrated in the figure, the loss function proposed in this paper demonstrates robustness in detecting various types of small PCB defects and exhibits terrific generalization capability.
(2) As shown in Table 4, the mAP values of Varifocal Loss are 5.4%, 4%, and 3.1% higher than those of Focal Loss, Quality Focal Loss, and Slide Loss, respectively.

## 4. Discussion

This paper proposes a lightweight PCB defect detection model, YOLO-BFRV, which builds upon the improved YOLOv8 architecture. To address the challenge of complex PCB backgrounds that hinder the model’s ability to extract various defective features effectively, the bidirectional feature pyramid network (BIFPN) is first incorporated to enhance feature extraction capabilities. Subsequently, FasterNet is integrated into the backbone network, improving the detection speed and accuracy while further reducing the model’s inference complexity. A re-parameterized detection header is then introduced to enhance the detection of small target defects while reducing computational parameters. Finally, the loss function is replaced with Varifocal Loss, further enhancing the model’s stability and improving its generalization ability. The proposed model also demonstrates excellent detection performance in the real-time monitoring of PCB defects, achieving an FPS of 142.5 and a mAP of 98.4%, which is 4.12% higher than the benchmark model YOLOv8s.

Although the YOLO-BFRV model proposed in this paper enhances the detection accuracy and speed of PCB defects, its performance on large-scale industrial public datasets requires further validation. When applied to actual production, the model demands high hardware performance, making it challenging to implement considering the cost and current working environment. Therefore, future research will focus on improving the model’s generalization and stability to enhance its effectiveness and practicality in industrial PCB defect detection applications.

## Figures and Tables

**Table 1:** Experimental environment configuration.

Programs	Settings
Operating system	Ubuntu
CPU	Intel(R) Xeon(R) Gold 6326 CPU @ 2.90GHz (Intel, Santa Clara, CA, USA)
GPU	NVIDIA A100 100GB PCIe (Nvidia, Santa Clara, CA, USA)
RAM	100 GB
Python version	3.7

**Table 2:** Experimental results of different models.

Model	mAP@50/%	P/%	R/%	Parameter Size
FasterR-CNN	84.5	87.3	92.7	24.59 M
YOLOv5	92.5	88.1	93.6	7.1 M
YOLOv7	94.7	89.3	94.3	37.1 M
YOLOv8s	94.5	92.6	95.1	30.2 M
RT-DETR	95.6	93.5	95.9	38.5 M
OURS	98.4	96.9	98.2	6.7 M

**Table 3:** Ablation experiments.

Model	BIFPN	FasterNet	RepHead	VarifocalLoss	mAP@50/%	P/%	R/%	fps/s	GFLOPS
**A**					94.5	92.6	95.1	97.4	6.3
**B**	✓				96.3	94	96.2	102.1	6.6
**C**		✓			97.4	96	95.6	134.2	10.7
**D**			✓		97.7	96.5	96.1	107.2	6.9
**E**				✓	95.6	94.6	93.8	98.6	6.3
**F**	✓	✓			97.4	95.9	96.6	138.5	8.2
**G**	✓		✓		96.5	94.6	97.2	118.2	8.6
**H**	✓			✓	96.9	96.4	95.2	109.2	7.1
**I**	✓	✓	✓	✓	98.4	96.9	98.2	142.1	11.5

**Table 4:** Results of loss function comparison experiments.

Loss Function	mAP50/%	P/%	R/%
Focal Loss	90.2	91.4	88.5
Quality Focal Loss	91.6	88.9	89.7
Slide Loss	92.5	90.6	90.5
Varifocal Loss	95.6	94.6	93.8

## Data Availability

The data are contained within the article.
